# Construct and criterion validity of the HiTOP spectra to predict dimensional and categorical somatization in a large non-western sample

**DOI:** 10.1038/s41598-023-40545-3

**Published:** 2023-08-14

**Authors:** Saeid Komasi, Azad Hemmati, Khaled Rahmani, Farzin Rezaei

**Affiliations:** 1 Neurosciences Research Center, Research Institute for Health Development, Kurdistan University of Medical Sciences, Sanandaj, Iran; 2 Department of Neuroscience and Psychopathology Research, Mind GPS Institute, Kermanshah, Iran; 3 Department of Psychology, University of Kurdistan, Sanandaj, Iran; 4 Liver and Digestive Research Center, Research Institute for Health Development, Kurdistan University of Medical Sciences, Sanandaj, Iran; 5 Department of Psychiatry, Roozbeh Hospital, Tehran University of Medical Sciences, Tehran, Iran

**Keywords:** Neuroscience, Psychology

## Abstract

The Hierarchical Taxonomy of Psychopathology (HiTOP) is a phenotypic data-driven framework for the classification of psychopathology. We tested the construct and criterion validity of the HiTOP spectra measured by the Personality Inventory for DSM-5 (PID-5) using exploratory structural equation modeling (ESEM) and hierarchical regressions both to predict somatic symptom and related disorders (SSRD) and a somatization factor. The case–control study used hierarchical logistic regressions to distinguish 257 cases with SSRD from 1007 healthy controls by both the maladaptive and the temperament factors. The extracted factors were also used in hierarchical linear regressions to predict the dimensional somatization factor. The seven temperament factors explained more variance above and beyond the five maladaptive factors when predicting SSRD (pseudo *R*^2^ = 0.169 to 0.266 versus 0.125 to 0.196; change in pseudo *R*^2^ = 0.055 to 0.087 versus 0.011 to 0.017). The temperament factors also explained more variance above and beyond the maladaptive factors when predicting the somatization factor (*R*^2^ = 0.392 versus 0.269; change in *R*^2^ = 0.146 versus 0.023). Although the HiTOP spectra measured by PID-5 are significant structures related to the categorical and dimensional measurements of somatoform, our findings highlight potential problems with both the construct and criterion validity of the HiTOP spectra.

## Introduction

The Hierarchical Taxonomy of Psychopathology (HiTOP) is a data-driven structural framework of mental symptoms created to enhance the reliability and validity of clinical utilization^1,2^. This structural data-based approach to the classification of general psychopathology uses factor analytical methods to cluster the mental symptoms into dimensional categories^3,4^. Compared to traditional diagnostic systems, HiTOP is a phenotypic model more consistent with both the genetic architecture of mental disorders and the impressions of environmental risk factors^5^ such as childhood abuse (i.e., the puzzle of parallel structure)^6,7^. This emerging classification system is also able to explain the long-term chronicity of psychopathology, account for functional impairment, and explain why disparate diagnoses from different classes respond to the same treatment patterns^5^.

Although there are some advantages over traditional diagnostic categories (e.g., use of dimensions), HiTOP remains a phenotypic model that organizes psychopathology based on symptom correlations (i.e., it takes a folk-taxonomy approach)^4,8^. This is a problem because of issues related to multifinality and equifinality. This means that mental symptoms do not always reflect etiology. It is possible for the same problem to be expressed by different symptoms, and for different problems to be expressed by overlapping symptoms. Thus, relying on symptom correlations may prevent a better understanding of the etiology of psychopathology, common etiological pathways responsible for the onset and progression of mental illness, and the dynamic developmental nature of psychopathological diagnoses^4,9^. At the conceptual level, HiTOP also implies “artefactual” comorbidity (e.g. patients with multiple disorders suffer from only one underlying condition) and a person-driven “snapshot” picture (e.g. self-reported symptoms at the time of investigation based on cross-sectional studies), and it neglects brain functional “perturbations” at the level of cell and systems. The instability of the higher-order structure over time, new inconsistencies caused by the extensions, and lack of explaining the rationale for the reintroduction of the somatoform diagnoses are considered at the methodological level^9^.

At first, HiTOP did not include the somatoform as a distinct category on the spectra level because there was not enough scientific evidence to support it^1^. However, recent research now provides additional empirical support for the somatoform as a distinct spectrum from the internalizing domain^10,11^. Despite the importance of the evidence, somatoform syndromes are not only differentially related to the gender and age group but are also associated with a wide range of spread and heterogeneous conditions in the HiTOP^9^. These issues and other problems with etiological pathways and genetic discovery are still considered serious challenges that complicate the implementation of the model, at least for somatoform diagnoses^4,9^.

In contrast to the HiTOP symptom-focused model, various etiological models for somatoform syndromes highlight the interplay between cognitive-perceptual processes and behavioral, affective, and biological variants^12^. Causal psychobiological mechanisms involved in somatoform syndromes include autonomic physiological arousal, the endocrine and immune systems, monoamine acids and neurotransmitters, brain mechanisms, and temperamental personality traits^12–17^. The association between some temperamental personality theories proposed by Akiskal et al.^18^, Cloninger^19,20^, and Lara et al.^21^, and somatoform diagnoses or Somatic Symptom and Related Disorders (SSRD) raised by the fifth edition of the Diagnostic and Statistical Manual of Mental Disorders (DSM-5) has already been investigated in some studies^14–17^. The five depressive, cyclothymic, hyperthymic, irritable, and anxious temperaments raised by Akiskal et al.^18^, are the affective components that were originally formulated for affective disorders. One study showed that cyclothymic, hyperthymic, irritable, and anxious temperaments are significantly related to somatization^16^. Cloninger's model suggests four temperament traits include novelty-seeking, harm avoidance, reward-dependence, and persistence^19,20^. Recent meta-analytic reviews have addressed strong associations between some temperaments such as harm avoidance and somatization^14,15^. Six emotional temperaments, including volition, anger, inhibition, sensitivity, control, and coping, along with twelve affective temperaments were components of Lara's dynamic theory^21^. A previous study reported associations between affective temperaments and somatic symptom severity in a large sample^17^.

Given that temperamental personality theories, unlike the HiTOP, derive from etiological theories that rely on neurobiological structures involved in the development, we aimed to investigate both the construct validity of the HiTOP spectra measured using the Personality Inventory for DSM-5 (PID-5) and the criterion validity of the somatoform domain in a large non-Western sample by a comparison between the HiTOP spectra measured using the PID-5 and the temperamental predispositions proposed by Akiskal et al.^18^, Cloninger^19,20^, and Lara et al.^21^. Because somatization is a complex psychiatric condition that is sometime introduced as an independent personality trait and that the dimensional personality assessment in patients with SSRD is widely ignored^22^, we first tried the extraction of a somatization factor (dimensional criterion variable) measured by various self-report instruments. We also tried to identify the higher-order factors of temperament traits measured by the Temperament Evaluation of Memphis, Pisa, Paris, and San Diego-Autoquestionnaire (TEMPS-A), the Temperament and Character Inventory (TCI), and the Affective and Emotional Composite Temperament Scale (AFECTS). Although there is little knowledge about the conjoint structure of temperaments and development in an integrated manner, personality traits are integrated within three disjoint genetic-environmental networks^23^.

The second step was taken to investigate the construct validity of the HiTOP spectra by identifying the higher-order factors of PID-5. The PID-5, which was originally designed to measure personality pathology^24^, is one of the instruments listed for measuring HiTOP constructs^1^. However, a dimensional personality disorder is strongly linked to the HiTOP structure. For example, a recent report addressed the role of the maladaptive personality domains assessed by PID-5 across HiTOP levels^25^. Another report attempts to address the placement of dimensional personality dysfunction within the HiTOP framework^26^. Although some studies in non-Western samples such as Iran have tried to test the construct validity of PID-5 maladaptive domains^27–30^, none of the studies included a large non-clinical sample as well as cases with SSRD^14,15^. Despite the present sample being from the west of Iran and this can make it difficult to generalize the results to other non-Western contexts, it is a unique effort to achieve advanced objectives. Finally, we tested the criterion validity of HiTOP by comparison between the maladaptive and temperament factors using hierarchical regressions to predict the somatization factor (dimensional approach) and differentiate cases with SSRD from healthy controls (categorical approach).

## Methods

### Design and context

This case–control study included 257 cases with SSRD (182 female; 70.8%) and 1007 HCs (648 female; 64.3%) from the west of Iran. The samples were selected from the Kermanshah and Sanandaj cities between April 2020 and August 2021. The population of these urban areas is approximately 1.5 million individuals, the majority of whom belong to the Kurdish ethnic group. All samples were individuals aged 18 and above, unmedicated for mental illnesses within the past month, and fluent in the Farsi language. Samples of the control group were selected from the general population by public announcements on popular platforms using convenient sampling. The initial general population encompassed 1900 college attendees, employees at health science facilities and other educational establishments, individuals seeking medical assistance, and housewives. Initially, 82.8% of people completed and returned the questionnaires (n = 1581). Questionnaires of 214 people also contained 15 to 90% of the missing data (n = 1367). One hundred and one subjects were excluded from the study due to physical symptoms of coronavirus (n = 57), physical problems from other medical conditions (n = 29), multiple sclerosis (n = 8), hepatitis (n = 4), severe epilepsy (n = 1), cancer (n = 1), and drug addiction (n = 1). Finally, the data of 1266 people were found to be usable.

In the following phase, individuals with SSRD were evaluated based on the self-reports of the subjects and specific threshold values (sensitive cutoff scores) for the Iranian population, which included scores exceeding 15.5 or 18 on the Screening for Somatic Symptom Disorders (SOMS-7) and the Short Health Anxiety Inventory (SHAI)^31,32^. Subsequently, a qualified clinical psychologist conducted an online diagnostic interview to examine the identified cases by the DSM-5 Criteria. Although 30 individuals were excluded due to their refusal to participate in the interview, the diagnosis of SSRD was verified for 229 individuals. As a result, the control group diminished from 1266 to 1007. Subsequently, we discovered 28 patients with SSRD from two psychiatric hospitals in Sanandaj and Kermanshah cities and appended them to the sample examined in the preceding phase (n = 28). Consequently, the number of cases with SSRD amounted to 257 individuals. All data and clinical interviews were carried out by two proficient psychologists. All subjects informed consent to participate in the study.

### Measures

The Personality Inventory for DSM-5 (PID-5) was used to evaluate the spectra on HiTOP. The PID-5 is a self-report dimensional questionnaire with 220 items to measure the pathological personality facets and five higher-order domains according to DSM-5 Section III^24^. Twenty five lower-order facets include emotional liability, separation insecurity, anxiousness, anhedonia, intimacy avoidance, withdrawal, grandiosity, manipulativeness, deceitfulness, irresponsibility, impulsivity, distractibility, perceptual dysregulation, unusual beliefs and experiences, eccentricity, risk-taking, callousness, attention-seeking, hostility, rigid perfectionism, perseveration, depressivity, submissiveness, restricted affectivity, and suspiciousness. The higher-order factors include negative affectivity, detachment, antagonism, disinhibition, and psychoticism. The domains are respectively equal to internalizing, detachment, antagonistic externalizing, disinhibited externalizing, and thought disorder on the HiTOP spectrum. Except for 16 items that are scored indirectly, each item is given a score between zero and three, from often false to often true. The higher scores on each domain or facet show a more severe pathology^24^. The PID-5 is a recommended instrument for measuring the HiTOP structure^1^, and its reliability and validity are acceptable in several Iranian samples^28–30^.

Temperaments were measured using three different instruments including the Temperament Evaluation of Memphis, Pisa, Paris, and San Diego-Autoquestionnaire (TEMPS-A), the Temperament and Character Inventory (TCI), and the Affective and Emotional Composite Temperament Scale (AFECTS). The original and short versions of TEMPS-A include 110 and 39 items, respectively^33^. The questionnaire measured five affective temperaments including depressive, cyclothymic, hyperthymic, irritable, and anxious traits. The Persian short version of TEMPS-A is formatted from 35 items for assessing depressive (8 items), cyclothymic (7 items), hyperthymic (8 items), irritable (6 items), and anxious (6 items) temperaments. All items of the questionnaire are scored directly (Yes = score 1, No = score 0), and the higher scores on each temperament trait (except hyperthymic) show more severe predispositions toward psychopathology. The Persian version of TEMPS-A is a reliable and validated instrument in the Iranian samples^34^.

The TCI is a dimensional self-report tool with versions of 56, 125, 140, and 240 items designed to measure four temperaments and three character traits^35^. Long forms of TCI are affected by cultural differences for Iranian samples^36^, and there is no official attempt to provide Iranian validations. Thus, we used the 125-item true–false version, which was well-standardized for Iranian culture with acceptable reliability and validity^37^. TCI-125 traits are novelty-seeking (20 items), harm avoidance (20 items), reward-dependence (15 items), persistence (5 items), self-directedness (25 items), cooperativeness (25 items), and self-transcendence (15 items). Sixty-one items in the questionnaire are scored directly (Yes = score 1, No = score 0) and 64 items are scored indirectly. Although the higher scores on the character components present more adaptive traits, very high/low scores on the temperament subscales show more severe predispositions toward psychopathology^35^. According to the objectives of the present study, we used only the temperament part.

The AFECTS is a two-part self-report scale with 60 items for dimensional measurements of the six emotional and twelve affective temperaments. The emotional section is a seven-point bipolar scale (score 0 to 7) for assessing volition (items 1–8), anger (items 9–16), inhibition (items 17–24), sensitivity (items 25–32), coping (items 33–40), and control (items 41–48). The emotional section is the personality dynamic part that tries to balance maladaptive predispositions (higher scores on anger, inhibition, and sensitivity) and adaptive predispositions (higher scores on volition, coping, and control). The affective section comprises internalizing (depressive, anxious, and apathetic), instable (cyclothymic, dysphoric, and volatile), stable (obsessive, euthymic, and hyperthymic), and externalizing (irritable, disinhibited, and euphoric) temperaments. Each item of the affective section is scored directly on a five-point Likert spectrum (from one = “it does not look like me at all” to five = “it looks exactly like me”) and the higher scores show a more severe predisposition toward psychopathology^21^. The Persian version of AFECTS is a reliable and validated instrument for the Iranian people^38^.

Somatization and illness anxiety were assessed by the Screening for Somatic Symptom Disorders-7 (SOMS-7), the Patient Health Questionnaire-15 (PHQ-15), the Revised Form of Symptom Checklist-90 (SCL-90-R), and the Short Health Anxiety Inventory (SHAI). The SOMS-7 is a self-report questionnaire with 47 items for evaluating the severity of the somatic signs/symptom during the recent week. The questionnaire consists of four subscales including pain, cardiovascular and respiratory symptoms (17 items), gastrointestinal and urologic symptoms (17 items), neurological functioning symptoms (10 items), and musculoskeletal symptoms (3 items). Each item is scored directly on a four-point Likert scale (from zero = never to three = always) and the higher scores on the total questionnaire or subscales present more severe somatic symptoms^39^. The SOMS-7 has acceptable validity and reliability among Iranian normal and clinical samples^31^.

The PHQ-15 is a self-report tool for measuring the severity of somatic symptoms during the last week. This questionnaire has no subscales and higher scores indicate more severe physical symptoms. Each of the items on the questionnaire is scored on a three-point scale from "not at all" (zero) to "a lot" (two) and the total score is ranged between zero and thirty. The reliability and validity of PHQ-15 have already been confirmed in Persian samples^40^.

The revised version of SCL-90 is a self-report questionnaire with 90 items for measuring mental symptoms. The nine subscales of the checklist include somatization, obsessive–compulsive disorder, depression, anxiety, hostility, phobic anxiety, interpersonal sensitivity, paranoid ideation, psychoticism, and six additional items. Each of the items scored on a five-point Likert scale from "no discomfort" (zero) to "very severe discomfort" (four) and the higher scores on the subscale or total questionnaire present a more severe mental symptomatology^41^. The Persian version of SCL-90-R is a reliable and validated instrument for the Iranian people^42^. In the present study, we used only the somatization subscale (12 items).

The SHAI is a self-report questionnaire with 18 items for assessing health anxiety. The three subscales of the questionnaire include rumination (7 items), probability of disease (7 items), and negative outcome (4 items). Each item of the SHAI is scored directly on a four-point Likert scale from "very low" (zero) to "very high" (three). The total score on the questionnaire is between zero and 54 and the higher scores show more severe health anxiety^32^. The reliability and validity of SHAI have already been confirmed in Persian samples^43^.

### Statistical analysis

In the first stage, we conducted a conjoint Exploratory Factor Analysis (EFA) with maximum likelihood estimations on four somatization measures including SOMS-7, PHQ-15, SCL-90-R somatization, and SHAI scores. Because we found a strong pattern of intercorrelations for both PID-5 and temperament measures, we test exploratory structural equation modeling (ESEM) to examine the factor structures of both the PID-5 and the temperament measures. ESEM is an effective approach that offers a confirmatory test of the early factor structures while allowing for the estimation of all cross-loadings^44,45^. We compared model fit for all factor solutions using Aikake Information Criteria (AIC), Bayesian Information Criteria (BIC), Root-Mean-Square Error of Approximation (RMSEA), Comparative Fit Index (CFI), and Tucker-Lewis Index (TLI) for four- to sex-factor solutions of the PID-5 as well as three- to eight-factor solutions of the temperament measures. A CFI equal to or greater than 0.96 and RMSEA equal to or less than 0.06 were acceptable for the select number of the factors^46,47^. We chose the parsimonious model including less latent factors when a change in CFI was less than or equal to 0.01^48^. Maximum likelihood estimations with Oblimin rotations performed by Mplus 7.4^49^ were used to select the traits/temperaments with the strongest loading on the factor-of-interest and the weakest loadings on all other factors.

In the next step, we used factor scores from ESEM analyses as predictors to construct two hierarchical linear regression models (somatization factor) and two hierarchical logistic regression models (SSRD group), in which the derived factors from both the PID-5 and temperament measures were alternatively entered as blocks to predict both the somatization factor and cases with SSRD, respectively. We compared the change in *R*^2^ to determine how much additional variance each model was explained in the outcome. The validity of the factors related to SSRD in each block was reported using Cox & Snell along with Nagelkerke (pseudo *R*^2^). The factors correlated to the somatization factor were identified using Beta statics and the factors related to SSRD were identified using Wald statics and an odd ratio (OR) with a 95% confidence interval (CI). All analyses were performed using SPSS software version 20 and a significance at the level of less than 0.05 was considered.

### Informed consent to participate and ethics approval

All subjects informed consent to participate in the study. This study is consistent with the Helsinki guidelines and it was approved by the ethics committee of the Kurdistan University of Medical Sciences (IR.MUK.REC.1398.169).

## Results

### Descriptive data and conjoint structure of somatization measures

The mean, standard deviation, skewness, kurtosis, and Cronbach's alpha of the somatization measures in the full sample (n = 1264) are seen in Appendix (Table S1). The conjoint structure of somatization measures was tested by EFA with maximum likelihood estimations. This analysis identifies a one-factor structure for somatization with eigenvalue = 2.71 which accounted for 68% of the common variance. The model with a one-factor solution showed acceptable goodness of fit (*χ*^2^ = 18.576, *p* < 0.001). The SOMS (= 0.867), PHQ (= 0.860), SCL90 somatization (= 0.831), and SHAI (= 0.444) respectively loaded more strongly on the extracted factor.

### Descriptive data and reliability of all personality/temperament measures

Both PID-5 and temperament measure statistics including the mean, standard deviation, skewness, kurtosis, and Cronbach's alpha of the scale items in the full sample are seen in Appendix (Tables S2 & S3). The mean score of all the PID–5 subscales was ranging from 0.70 (callousness) to 1.30 (risk-taking) while the mean score of all the temperament subscales was ranging from 1.40 (TEMPS-A anxious) to 37.03 (AFECTS coping). Cronbach’s alpha of all the PID–5 subscales were ranging from 0.51 (suspiciousness) to 0.91 (depressivity and eccentricity), while Cronbach’s alpha of all the temperament subscales were ranging from 0.43 (TCI reward dependence) to 0.91 (AFECTS volition).

### Conjoint structure of temperament measures

We compared three ESEM models four- to eight-factor solutions to test the structure of the temperament measures, the results of which are shown in Table 1. We chose seven-factor solutions to interpret the conjoint structure of temperament because it is a well-fitting and more parsimonious extracted model (RMSEA ≤ 0.05, CFI = 0.96, all* p* < 0.001, change in CFI > 0.01). Table 2 shows temperament factor loadings, eigenvalues, and factor correlations of the seven latent factors. Temperament factors include Factor I (instability: high cyclothymic temperament and reward-dependence), Factor II (negative affectivity: high anxious, irritable, and depressive), Factor III (positive emotionality: high coping, volition, and control), Factor IV (negative emotionality: high anger, sensitivity, and inhibition), Factor V (internalization: high anxious, depressive, and harm avoidance along with low novelty seeking), Factor VI (externalization: high euphoric, disinhibited, irritable, cyclothymic, volatile, dysphoric, and apathetic), and Factor VII (stability: high hyperthymic, euthymic, obsessive, and persistence). All cross-factor coefficients were between − 0.40 and 0.90 while factor intercorrelations were between − 0.31 and 0.48. Totally, the factors could explain 51% of the common variance.

**Table 1:** ESEM model fit comparisons for the PID-5 and the temperament measures.

Personality systems	χ^2^ (df)	CFI	TLI	RMSEA [90% CI]	AIC	BIC
The PID-5						
Four factors	1486.27 (206)	0.94	0.92	0.07 [0.07, 0.07]	167,693	168,433
Five factors	1008.64 (185)	0.96	0.94	0.06 [0.06, 0.06]	167,257	168,106
Six factors	754.93 (165)	0.97	0.95	0.05 [0.05, 0.06]	167,043	167,995
Temperament						
Four factors	1934.12 (249)	0.88	0.83	0.07 [0.07, 0.08]	138,324	137,829
Five factors	1334.23 (226)	0.92	0.88	0.06 [0.06, 0.07]	136,968	137,889
Six factors	1008.26 (204)	0.94	0.90	0.06 [0.05, 0.06]	136,686	137,720
Seven factor	701.55 (183)	0.96	0.93	0.05 [0.04, 0.05]	136,421	136,858
Eight factor	550.61 (163)	0.97	0.94	0.04 [0.04, 0.05]	136,310	137,555

CFI: Comparative Fit Index; TLI: Tucker-Lewis Index; RMSEA: root mean square error of approximation; AIC: Akaike’s information criteria; BIC: Bayesian information criteria.

**Table 2:** Temperament factor loadings and factor correlations.

Temperament traits	Factor						
	I	II	III	IV	V	VI	VII
TEMPS-A cyclothymic	**0.62**	0.26	− 0.00	− 0.03	− 0.07	0.09	0.10
TCI reward dependence	**0.44**	− 0.28	0.03	0.02	− 0.03	− 0.05	− 0.10
TEMPS-A anxious	− 0.05	**0.82**	− 0.02	0.00	− 0.09	0.07	− 0.04
TEMPS-A irritable	0.14	**0.73**	− 0.07	0.03	0.12	− 0.05	− 0.03
TEMPS-A depressive	0.29	**0.46**	− 0.09	0.03	0.21	0.04	− 0.14
AFECTS coping	− 0.01	− 0.03	**0.90**	− 0.05	0.03	− 0.01	0.03
AFECTS volition	− 0.04	− 0.12	**0.67**	− 0.09	− 0.19	0.08	0.10
AFECTS control	0.06	0.04	**0.62**	0.25	0.06	− 0.05	0.07
AFECTS anger	− 0.05	0.05	− 0.17	**0.63**	− 0.10	0.23	0.03
AFECTS sensitivity	0.08	− 0.04	0.39	**0.49**	− 0.01	− 0.03	− 0.15
AFECTS inhibition	− 0.01	0.06	0.39	**0.43**	0.25	− 0.10	− 0.08
AFECTS anxious	0.09	0.14	− 0.08	0.03	**0.61**	0.14	0.02
AFECTS depressive	− 0.07	0.28	− 0.01	− 0.08	**0.44**	0.26	− 0.12
TCI novelty seeking	0.27	0.08	− 0.07	− 0.00	** − 0.40**	0.35	− 0.07
TCI harm avoidance	0.19	0.04	− 0.19	0.17	**0.34**	− 0.06	− 0.32
AFECTS euphoric	− 0.01	0.05	0.03	0.07	− 0.09	**0.68**	0.05
AFECTS disinhibited	0.06	0.04	− 0.02	0.01	0.05	**0.61**	− 0.01
AFECTS irritable	− 0.06	0.10	− 0.04	0.12	0.06	**0.55**	0.23
AFECTS cyclothymic	0.30	− 0.04	− 0.04	0.01	0.20	**0.46**	− 0.12
AFECTS volatile	0.21	0.07	− 0.07	0.03	0.21	**0.44**	− 0.15
AFECTS dysphoric	0.27	0.02	− 0.10	0.10	0.29	**0.35**	− 0.03
AFECTS apathetic	− 0.07	0.22	− 0.03	− 0.10	0.24	**0.32**	− 0.30
AFECTS hyperthymic	0.02	− 0.10	0.06	− 0.01	− 0.01	0.05	**0.75**
AFECTS euthymic	− 0.08	− 0.13	0.09	− 0.13	0.12	− 0.07	**0.53**
AFECTS obsessive	0.04	0.03	0.03	0.08	0.26	0.07	**0.46**
TEMPS-A hyperthymic	0.09	0.16	0.12	− 0.08	− 0.30	0.09	**0.43**
TCI persistence	0.10	0.12	0.03	0.09	0.13	− 0.21	**0.38**
Factor correlation							
F2	0.41						
F3	− 0.16	− 0.38					
F4	0.20	0.15	0.17				
F5	0.21	0.31	− 0.14	0.21			
F6	0.36	0.48	− 0.27	0.13	0.13		
F7	− 0.19	− 0.26	0.39	− 0.18	− 0.31	− 0.04	
Initial eigenvalues	7.50	2.74	2.35	1.48	1.19	1.10	0.98
% of variance	27.79	10.15	8.69	5.46	4.41	4.09	3.62
Cumulative %	27.79	37.94	46.63	52.09	56.50	60.59	64.21
Squared eigenvalues	6.98	2.45	1.88	0.79	0.80	0.57	0.52
% of variance	25.84	8.33	6.95	2.93	2.98	2.10	1.91
Cumulative %	25.84	34.17	41.12	44.06	47.04	49.14	51.06

The strongest factor loadings are shown in bold.

AFECTS: Affective and Emotional Composite Temperament Scale; TEMPS-A: Temperament Evaluation of Memphis, Pisa, Paris, and San Diego Autoquestionnaire; TCI: temperament and character inventory.

### Conjoint structure of maladaptive domains (construct validity of the spectra on HiTOP)

We compared three ESEM models four- to sex-factor solutions to test the structure of the PID-5, the results of which are shown in Table 1. We chose five-factor solutions to interpret the PID-5 because it is a well-fitting and more parsimonious extracted model (RMSEA ≤ 0.06, CFI = 0.96, all* p* < 0.001, change in CFI > 0.01). Table 3 shows PID-5 factor loadings, eigenvalues, and factor correlations of the five latent factors. The maladaptive factors include Factor I (negative affectivity: anxiousness, depressivity, distractibility, anhedonia, emotional liability, impulsivity, perseveration, submissiveness, separation insecurity, and suspiciousness), Factor II (antagonism: deceitfulness, callousness, manipulativeness, irresponsibility, and risk-taking), and Factor III (detachment: restricted affectivity, withdrawal, and intimacy avoidance), Factor IV (thought disorder: unusual beliefs and experiences, eccentricity, and perceptual dysregulation) and Factor V (narcissism: attention-seeking, grandiosity, rigid perfectionism, and hostility). All cross-factor coefficients were between − 0.28 and 0.82 while factor intercorrelations were between 0.18 and 0.63. Totally, the factors could explain 63% of the common variance.

**Table 3:** PID-5 factor loadings and factor correlations.

PID-5 facets	Factor				
	I	II	III	IV	V
Anxiousness	**0.71**	− 0.11	0.12	0.07	0.11
Depressivity	**0.65**	0.14	0.19	0.17	− 0.13
Distractibility	**0.63**	0.16	0.11	0.06	0.10
Anhedonia	**0.62**	0.08	0.37	− 0.04	− 0.15
Emotional liability	**0.57**	− 0.02	− 0.15	0.26	0.35
Impulsivity	**0.51**	0.38	− 0.20	0.15	− 0.00
Perseveration	**0.46**	0.01	0.24	0.18	0.29
Submissiveness	**0.42**	− 0.03	0.12	− 0.01	0.28
Separation insecurity	**0.41**	0.20	− 0.02	0.09	0.26
Suspiciousness	**0.29**	0.02	0.17	0.17	0.22
Deceitfulness	0.10	**0.75**	0.04	0.10	0.06
Callousness	0.01	**0.72**	0.20	0.36	-0.10
Manipulativeness	− 0.19	**0.66**	0.08	0.14	− 0.02
Irresponsibility	0.23	**0.63**	0.05	0.13	− 0.13
Risk-taking	− 0.15	**0.39**	− 0.28	0.33	0.04
Restricted affectivity	− 0.00	0.16	**0.65**	0.09	0.13
Withdrawal	0.17	0.09	**0.61**	0.14	0.04
Intimacy avoidance	0.02	0.09	**0.53**	0.13	− 0.13
Unusual beliefs	− 0.08	0.03	0.07	**0.82**	0.04
Eccentricity	0.19	0.16	0.11	**0.57**	0.03
Perceptual dysregulation	0.32	0.17	0.07	**0.56**	− 0.07
Attention seeking	0.18	0.24	− 0.09	− 0.05	**0.66**
Grandiosity	− 0.24	0.19	0.10	0.25	**0.59**
Rigid perfectionism	0.17	− 0.27	0.24	0.22	**0.57**
Hostility	0.36	0.34	0.12	− 0.06	**0.36**
Factor correlation					
F2	0.35				
F3	0.43	0.27			
F4	0.46	0.63	0.45		
F5	0.26	0.27	0.18	0.42	
Initial eigenvalues	11.76	2.17	1.64	1.29	0.80
% of variance	47.04	8.67	6.54	5.16	3.21
Cumulative %	47.04	55.72	62.25	67.41	70.63
Squared eigenvalues	11.40	1.78	1.28	0.90	0.43
% of variance	45.60	7.11	5.10	3.59	1.72
Cumulative %	45.60	52.70	57.80	61.39	63.11

The strongest factor loadings are shown in bold.

PID-5: Personality Inventory for DSM-5.

### Prediction of the dimensional somatization (criterion validity of the spectra on HiTOP)

Table 4 contains the hierarchical linear regression models for determining factors related to the somatization factor. When the five maladaptive factors were entered in the first block, the *R*^2^ of the model was 0.269 (*p* < 0.001), whereas when the seven temperament factors were entered in the first block, the *R*^2^ of the model was 0.392 (*p* < 0.001). As such, our main focus is on the relative change in *R*^2^ values for models with the somatization factor as the dependent variable. The temperament factors were explained more variance above and beyond the maladaptive factors when predicting the somatization factor (change in *R*^2^ = 0.146, *p* < 0.001). Conversely, the maladaptive factors were explained less variance above and beyond the temperament factors (change in *R*^2^ = 0.023, *p* < 0.001). Overall, all the factors could predict a 41.5% variance of the somatization factor). We also report standardized Beta coefficients from each of the specific factors, which the results also can be seen in Table 4. According to the Beta coefficients reported in this Table, two maladaptive factors (*β* =  − 0.270 and 0.335*, p* < 0.001) and two temperament factors (*β* = 0.111 and 0.534*, p* < 0.001) are significantly related to the somatization factor. Therefore, the factors of both systems are complementary to predict the somatization factor.

**Table 4:** The hierarchical linear regressions for determining factors related to the somatization factor.

Factors	Block 1 (separated)			Block 2 (integrated)		
	Beta	Std. error	*P*	Beta	Std. error	*P*
PID-5						
I	0.633	0.025	< 0.001	0.099	0.027	0.283
II	− 0.226	0.032	0.001	− 0.270	0.030	< 0.001
III	− 0.121	0.017	0.015	− 0.034	0.016	0.463
IV	0.325	0.028	0.001	0.335	0.027	< 0.001
V	− 0.188	0.030	0.003	− 0.131	0.030	0.039
*R*^2^	0.269		< 0.001	0.023		< 0.001
Temperament						
I	− 0.052	0.002	0.031	− 0.030	0.002	0.214
II	0.516	0.029	< 0.001	0.534	0.030	< 0.001
III	0.087	0.007	0.159	0.074	0.007	0.226
IV	− 0.073	0.019	0.119	− 0.071	0.019	0.132
V	0.291	0.146	0.003	0.170	0.148	0.088
VI	− 0.057	0.088	0.314	− 0.010	0.094	0.875
VII	0.104	0.045	0.010	0.111	0.046	0.007
*R*^2^	0.392		< 0.001	0.146		< 0.001

Cumulative *R*^2^ = 0.415.

PID-5: Personality Inventory for DSM-5.

### Prediction of the categorical somatization (criterion validity of the spectra on HiTOP)

Table 5 shows hierarchical logistic regression models to determine factors related to SSRD. When the five maladaptive factors were entered in the first block, the pseudo *R*^2^ of the model was 0.125 to 0.196 (*p* < 0.001), whereas when the seven temperament factors were entered in the first block, the pseudo *R*^2^ of the model was 0.169 to 0.266 (*p* < 0.001). As such, our main focus is on the relative change in *R*^2^ values for models with SSRD as the dependent variable. The temperament factors were explained more variance above and beyond the maladaptive factors when predicting SSRD (change in pseudo *R*^2^ = 0.055 to 0.087, *p* < 0.001). Conversely, the maladaptive factors were explained less variance above and beyond the temperament factors (change in pseudo *R*^2^ = 0.011 to 0.017, *p* = 0.005). Overall, all the factors predict an 18 to 28.3% variance of SSRD. We also report Wald statistics and odd ratio (OR) with a 95% confidence interval (CI) for each of the specific factors, which the results also can be seen in Table 5. According to the Wald statistics reported in this Table, one maladaptive factor (OR = 1.30*, p* = 0.009) and the two temperament factors (OR = 4.35 and 1.29, *p* < 0.001) are significantly related to SSRD. Therefore, the factors of both systems are complementary to differentiate cases with SSRD from healthy controls.

**Table 5:** The hierarchical logistic regressions for determining factors related to SSRD.

Factors	Block 1 (separated)			Block 2 (integrated)		
	Wald	OR (95% CI)	P	Wald	OR (95% CI)	P
PID-5						
I	14.95	1.37 (1.17, 1.60)	< 0.001	0.62	0.92 (0.75, 1.13)	0.430
II	0.04	1.02 (0.84, 1.24)	0.849	0.64	0.92 (0.74, 1.14)	0.425
III	0.70	0.95 (0.86, 1.07)	0.402	0.80	0.95 (0.84, 1.07)	0.372
IV	2.29	1.15 (0.96, 1.37)	0.130	6.90	1.30 (1.07, 1.58)	0.009
V	4.11	0.81 (0.66, 0.99)	0.043	0.01	0.99 (0.78, 1.24)	0.906
*R*^2^	0.125–0.196		< 0.001	0.011–0.017		0.005
Temperament						
I	0.35	1.00 (0.99, 1.02)	0.555	0.17	1.00 (0.99, 1.01)	0.677
II	11.29	1.40 (1.15, 1.70)	0.001	5.99	1.29 (1.05, 1.57)	0.014
III	0.00	1.00 (0.95, 1.06)	0.981	0.18	0.99 (0.94, 1.04)	0.675
IV	0.37	0.96 (0.84, 1.10)	0.545	0.01	0.99 (0.86, 1.14)	0.926
V	6.91	3.80 (1.41, 10.30)	0.009	7.80	4.35 (1.56, 12.19)	0.005
VI	1.48	0.68 (0.37, 1.26)	0.224	3.59	0.52 (0.27, 1.02)	0.058
VII	1.20	1.20 (0.87, 1.66)	0.272	1.01	1.19 (0.85, 1.66)	0.314
*R*^2^	0.169–0.266		< 0.001	0.055–0.087		< 0.001

*R*^2^ is following the Cox & Snell–Nagelkerke indices; *R*^2^: cumulative validity = 0.180–0.283.

OR: odds ratio, PID-5: Personality Inventory for DSM-5, SSRD: somatic symptom and related disorders.

## Discussion

We examined the validity of HiTOP by comparison between the maladaptive and temperament factors, both to predict SSRD and the somatization factor. Unlike temperamental theories, the HiTOP is a phenotypic (i.e., symptom-based) classification system that is agnostic to neurobiological pathways and other etiological causal mechanisms related to psychopathology. The somatoform spectrum on HiTOP also relies mainly on findings based on the DSM-IV diagnostic criteria or earlier versions of DSM for somatoform diagnoses and similar categories such as hypochondriasis^1,10^. Although more recent studies attempt to restore the validity of HiTOP and useful tools using the SSRD raised by the DSM-5^11,22,50^, convincing cross-cultural empirical evidence with a prospective longitudinal approach in large populations, especially non-Western samples, is not yet available. These considerations highlight the necessity of validating the HiTOP model as currently, there is insufficient evidence to introduce the somatoform as an independent spectrum on HiTOP^1,9^.

The purpose of the current study was to further investigate both the construct and criterion validity of the HiTOP spectra. This is important not only for testing the validity of HiTOP but also because the validation of psychological models helps to improve the illness screening system and provide appropriate interventions to prevent more serious outcomes. The validation of models can be done internally using the same source or comparing explanations with other models in new contexts and externally using new data from an independent data set^51^. The difficulty for generalizability is the most important limitation of the developed models, which can be well tested using construct validation across cultures before clinical application^51,52^. We assessed both construct and criterion validity using factor analysis and regression techniques. Although factor analysis and ESEM techniques test construct validity by identifying strongly correlated latent factors in a dataset, the higher-order common factors only characterize the phenotypic aspects of psychopathology. Such a basis cannot provide accurate insight into the effects of genotypic and environmental factors on the incidence and development of psychiatric disorders such as somatization.

Our results showed that PID-5 facets loaded on five independent higher-order factors including the components of negative affectivity or internalizing (Factor I), antagonistic externalizing (Factor II), detachment (Factor III), thought disorder (Factor IV), and narcissism (Factor V). Although factors I to IV cover all domains of the HiTOP spectra, the fifth extracted factor do not correspond to the five independent factors introduced by both the PID-5 and HiTOP. Not the disinhibition factor but the narcissism factor including attention seeking, grandiosity, rigid perfectionism, and hostility was identified. This finding was not consistent with the results of recent meta-analyses that include the disinhibition factor^27,53^. However, recent studies in Iran showed that disinhibition traits tend to load on the negative affectivity and antagonism factors^28,29^. In our study, some disinhibited traits including distractibility and impulsivity were strongly loaded on the negative affectivity factor, while irresponsibility and risk-taking were loaded on the antagonism factor. The narcissism factor containing anankastic (rigid perfectionism) and antagonistic (attention seeking and grandiosity) traits is somewhat similar to the results of the previous report in the samples of western Iran^28^. Therefore, we conclude that the replicability of the maladaptive domains of the PID-5 and the HiTOP spectra in the present non-Western sample is somewhat questionable. Despite supporting the five-factor structure by ESEM, not extracting the disinhibition factor independently can indicate the challenges of the construct validity of spectra on the HiTOP. However, the difference in factor loads is probably due to the estimation and rotation methods^54^.

We also aimed to identify higher-order temperament factors of the 27 traits proposed by three independent theories^18–21^. Our findings showed that temperament traits, loaded on seven independent higher-order factors, including instability (Factor I), negative affectivity (Factor II), positive emotionality (Factor III), negative emotionality (Factor IV), internalization (Factor V), externalization (Factor VI), and stability (Factor VII). Previous findings support the hierarchical structure of temperaments proposed in different theories^55,56^. Although we could not find studies to identify the higher-order factors of the three proposed theories, a recent report showed that temperament and character traits are integrated within some unique genetic-environmental networks^23^.

The findings of the present study show that the higher-order factors extracted from PID-5 and temperament predispositions can potentially complement each other in the categorical and dimensional measurement of the somatoform spectrum on HiTOP. This finding supports the discriminant validity of both maladaptive and temperament factors. However, the incremental validity of maladaptive factors was much smaller than the temperament factors. In more detail, the PID-5 maladaptive factors in both the categorical and dimensional assessments of somatization were able to improve the temperament factors by only two percent. Although the partial incremental contribution was statistically significant, it was much smaller than the incremental validity of the temperament factors (i.e., about 6 to 15%). Although we know that adding more predictors to a regression model may provide more explanation for the variance, we aimed to compare maladaptive and temperament factors to identify a more efficient framework.

These findings underscore potential problems with the criterion validity of the HiTOP spectra, at least about the intercorrelations with the somatoform spectrum. Of course, it should be noted that it is not practically possible to measure all spectra structures or other HiTOP levels even with recommended tools such as PID-5^1^. The PID-5 facets are less involved in the underlying psychopathology of somatoform disorders, including attachment style, emotional awareness and dysregulation, and alexithymia^57–61^. Although emotional dysfunction was recently introduced as a higher-order factor covering somatoform and internalizing domains^10^, the PID-5 only includes a few underlying and revealing facets of the somatoform such as emotional liability, separation insecurity, anxiousness, perceptual dysregulation, restricted affectivity, and distractibility (low)^59–63^. Following such problems, recent efforts have been directed to design practical tools with the ability to measure various structures on all levels of HiTOP^50,64^. But unlike the recommended tools for measuring HiTOP structures, temperament theories and tools, especially emotional and affective temperaments, are completely dependent on emotional structures predisposing to psychopathology^18,21^. The results of the present study indicated that both the negative affectivity factor and internalization factor of temperament are facilitators of both somatization and SSRD. Some previous evidence also supports the key role of emotional and affective temperaments in SSRD and somatization^16,17,65^. Our conjoint factor analytic measures are useful for testing the cross-cultural generalizability of the extracted constructs. However, the present research do not have the merits for cross-cultural studies of etiology and development that are based on measures of individual differences in biological and genetic variables that are shared by all humans across cultures and environments, as validated by TCI temperaments^66,67^.

Our work with a sectional case–control study and the large sample size is unique, at least in a non-Western context. Indeed, The target populations in most clinical psychology studies are white people from the West^68^. Thus, the current study provides much-needed diversification to the research field. However, we only included samples from western Iran (mainly Kurdish culture), which makes it difficult to generalize the findings to other regions of Iran or other non-Western contexts. We tried to achieve advanced findings about a more comprehensive classification of temperaments by identifying higher-order temperament factors proposed in three independent theories^18–21^. Dimensional and categorical measurement of somatoform in the role of criterion variable provided the possibility of independent analyses, and similar findings related to both assessments supported the valid methodology and analysis of the present study. We reported the internal consistency of all questionnaires and found all of them to be reasonably reliable. This supports the validity of the assessment and reduced bias caused by the self-report instruments used in the present cross-sectional study. Conversely, using one tool such as PID-5 to evaluate the constructs of a model such as HiTOP is not enough, and validation of the psychological models requires the use of more comprehensive tools^1,50,64^. Using self-report questionnaires at one point in time only provides a person-driven “snapshot” picture^9^, while prospective longitudinal studies to investigate the external temporal validation^51^ can provide valuable information. Although comorbidity cannot confound the results related to the somatoform dimensional measurement, it may potentially affect the findings obtained from the categorical assessment of the case–control study. Finally, the gender distribution of the sample in the two groups was somewhat unequal. The lack of statistical significance of this difference should not mislead us and we recommend gender matching in future studies.

In sum, evidence from the present study supports a five-factor structure relatively similar to the PID-5 containing a narcissism factor instead of a disinhibition factor. This indicates a relatively weak construct validity, which, in addition to culture, can be partially influenced by the estimation and rotation methods. These results also raise further questions about the generalizability of HiTOP to people from the non-Western world. The HiTOP framework is derived almost exclusively from data from American and European samples, and thus, may not generalize (or it may even promote systematic bias) to other cultures and underrepresented groups^4^. We found that the spectra on HiTOP measured by PID-5 are significant structures related to both the categorical and dimensional measurements of the somatization. Our findings also support the discriminant validity of both maladaptive and temperament higher-order factors as potentially complementary structures associated with the SSRD. Nevertheless, the incremental validity of maladaptive factors to both the categorical and dimensional somatization was much smaller than the temperament factors. These results highlight potential problems with the criterion validity of the HiTOP spectra, at least about the intercorrelations with the somatoform spectrum. Future studies considering the methodological limitations of the present study can provide more evidence for the validity of the spectra on HiTOP.

### Supplementary Information

Supplementary Tables.

## Data Availability

Data will be made available upon request from the corresponding author.
