# Supplementary material for: Construct and criterion validity of the HiTOP spectra to predict dimensional and categorical somatization in a large non-western sample
Source: Sci Rep. 2023 Aug 14;13:13197. doi: 10.1038/s41598-023-40545-3 (PMC10425466; doi:10.1038/s41598-023-40545-3)
Supplement: Supplementary file 1 — Supplementary Tables. [file 41598_2023_40545_MOESM1_ESM.doc]

**Table S1.** The statistics of somatization measures (n = 1264)

| Somatization scale | *N* item | *α* | Mean | SD | Skewness | Kurtosis |
| --- | --- | --- | --- | --- | --- | --- |
| SOMS-7 | 47 | .94 | 17.93 | 16.40 | 1.43 | 1.93 |
| SCL-90-R | 12 | .90 | 13.12 | 9.91 | .78 | .01 |
| PHQ-15 | 15 | .84 | 27.15 | 5.21 | 1.05 | 1.30 |
| SHAI | 18 | .84 | 17.08 | 8.28 | .63 | .23 |

Abbreviation_ SOMS-7: Screening for Somatic Symptom Disorders-7, PHQ-15: Patient Health Questionnaire-15, SCL-90-R: Symptom Checklist-90-Revised Form, SHAI: Short Health Anxiety Inventory, SD: Standard deviation.

**Table S2.** The PID-5 trait statistics (n = 1264)

| Maladaptive traits | *N* item | *α* | Mean | SD | Skewness | Kurtosis |
| --- | --- | --- | --- | --- | --- | --- |
| Emotional liability | 7 | .76 | 1.16 | .62 | .13 | − .48 |
| Anxiousness | 9 | .85 | 1.14 | .66 | .44 | − .33 |
| Separation insecurity | 7 | .76 | .94 | .62 | .33 | − .57 |
| Withdrawal | 10 | .83 | .92 | .57 | .21 | − .46 |
| Anhedonia | 8 | .77 | 1.04 | .58 | .29 | − .53 |
| Intimacy avoidance | 6 | .68 | .98 | .60 | .39 | − .20 |
| Manipulativeness | 5 | .61 | .84 | .55 | .52 | .06 |
| Deceitfulness | 10 | .82 | .77 | .56 | .69 | − .02 |
| Grandiosity | 6 | .70 | 1.10 | .58 | .12 | − .50 |
| Irresponsibility | 7 | .72 | .76 | .54 | .62 | − .22 |
| Impulsivity | 6 | .82 | .87 | .67 | .61 | − .21 |
| Distractibility | 9 | .83 | .95 | .60 | .30 | − .36 |
| Unusual beliefs | 8 | .77 | .79 | .57 | .46 | − .50 |
| Eccentricity | 13 | .91 | .77 | .63 | .57 | −.42 |
| Perceptual dysregulation | 12 | .83 | .71 | .52 | .49 | − .41 |
| Attention seeking | 8 | .80 | 1.17 | .61 | .15 | − .36 |
| Callousness | 14 | .85 | .70 | .52 | .79 | .05 |
| Depressivity | 14 | .91 | .78 | .63 | .59 | − .44 |
| Hostility | 10 | .81 | 1.08 | .58 | .13 | − .45 |
| Perseveration | 9 | .79 | 1.04 | .56 | −.02 | − .62 |
| Restricted affectivity | 7 | .67 | .99 | .54 | .27 | − .46 |
| Rigid perfectionism | 10 | .75 | 1.27 | .54 | − .05 | − .03 |
| Risk-taking | 14 | .69 | 1.30 | .42 | .14 | .49 |
| Submissiveness | 4 | .56 | 1.10 | .60 | .13 | − .47 |
| Suspiciousness | 7 | .51 | 1.28 | .48 | .32 | .10 |

Abbreviation_ PID-5: Personality Inventory for DSM-5, SD: Standard deviation.

**Table S3.** Temperament measure statistics (n = 1264)

| Temperament traits | *N* item | *α* | Mean | SD | Skewness | Kurtosis |
| --- | --- | --- | --- | --- | --- | --- |
| TEMPS-A Depressive | 8 | .80 | 2.77 | 2.42 | .52 | − .90 |
| TEMPS-A Cyclothymic | 7 | .51 | 3.37 | 1.73 | .06 | − .64 |
| TEMPS-A Hyperthymic | 8 | .59 | 4.44 | 1.92 | −.14 | − .63 |
| TEMPS-A Irritable | 6 | .71 | 1.88 | 1.76 | .60 | −.75 |
| TEMPS-A Anxious | 6 | .62 | 1.40 | 1.44 | 1.02 | .40 |
| TCI Novelty seeking | 20 | .63 | 9.01 | 3.33 | .25 | −.22 |
| TCI Harm avoidance | 20 | .78 | 8.78 | 4.27 | .19 | −.46 |
| TCI Reward dependence | 15 | .43 | 8.28 | 2.30 | .04 | −.25 |
| TCI Persistence | 5 | .51 | 3.15 | 1.38 | − .54 | −.47 |
| AFECTS Volition | 8 | .91 | 36.76 | 10.94 | −.29 | − .28 |
| AFECTS Anger | 8 | .83 | 28.49 | 9.37 | .10 | − .26 |
| AFECTS Inhibition | 8 | .62 | 32.83 | 7.04 | −.30 | .67 |
| AFECTS Sensitivity | 8 | .59 | 29.13 | 5.95 | −.18 | 1.05 |
| AFECTS Coping | 8 | .89 | 37.03 | 10.30 | −.32 | −.10 |
| AFECTS Control | 8 | .59 | 34.48 | 6.91 | −.39 | .87 |
| AFECTS Depressive | 1 | - | 1.99 | 1.19 | 1.01 | .10 |
| AFECTS Anxious | 1 | - | 2.39 | 1.21 | .43 | −.80 |
| AFECTS Apathetic | 1 | - | 1.99 | 1.09 | .87 | − .11 |
| AFECTS Cyclothymic | 1 | - | 2.63 | 1.21 | .21 | − .84 |
| AFECTS Dysphoric | 1 | - | 2.62 | 1.09 | .09 | − .60 |
| AFECTS Volatile | 1 | - | 2.26 | 1.14 | .62 | − .42 |
| AFECTS Obsessive | 1 | - | 3.09 | 1.06 | − .19 | − .44 |
| AFECTS Euthymic | 1 | - | 3.24 | 1.07 | −.30 | −.50 |
| AFECTS Hyperthymic | 1 | - | 3.19 | 1.13 | −.17 | − .61 |
| AFECTS Irritable | 1 | - | 2.53 | 1.09 | .29 | − .54 |
| AFECTS Disinhibited | 1 | - | 2.35 | 1.07 | .45 | − .43 |
| AFECTS Euphoric | 1 | - | 2.32 | 1.11 | .54 | − .44 |

Abbreviations_ AFECTS: Affective and Emotional Composite Temperament Scale, TEMPS-A: Temperament Evaluation of Memphis, Pisa, Paris, and San Diego Autoquestionnaire, TCI: Temperament and Character Inventory, SD: Standard deviation.
